# Serum Trimethylamine *N*-Oxide Level Is Positively Associated with Aortic Stiffness Measured by Carotid–Femoral Pulse Wave Velocity in Patients Undergoing Maintenance Hemodialysis

**DOI:** 10.3390/toxins15090572

**Published:** 2023-09-17

**Authors:** Po-Yu Huang, Bang-Gee Hsu, Yu-Hsien Lai, Chih-Hsien Wang, Jen-Pi Tsai

**Affiliations:** 1 Division of Nephrology, Department of Internal Medicine, Dalin Tzu Chi Hospital, Buddhist Tzu Chi Medical Foundation, Chiayi 62247, Taiwan; poyuhs13628@gmail.com; 2 Division of Nephrology, Hualien Tzu Chi Hospital, Buddhist Tzu Chi Medical Foundation, Hualien 97004, Taiwan; gee.lily@msa.hinet.net (B.-G.H.); hsienhsien@gmail.com (Y.-H.L.); wangch33@gmail.com (C.-H.W.); 3 School of Medicine, Tzu Chi University, Hualien 97004, Taiwan

**Keywords:** carotid–femoral pulse wave velocity, trimethylamine *N*-oxide, hemodialysis, aortic stiffness

## Abstract

Trimethylamine *N*-oxide (TMAO) is a biomarker that is effective in predicting major adverse cardiovascular (CV) events. Age-related vascular problems are significantly affected by aortic stiffness (AS), which is independently linked to CV morbidity and mortality. This study aimed to determine the association between serum TMAO levels and carotid–femoral pulse wave velocity (cfPWV) in patients receiving hemodialysis (HD) therapy. In total, 115 patients with HD were enrolled in this study. The AS group included patients whose cfPWV was >10 m/s. Using high-performance liquid chromatography and mass spectrometry, the levels of serum TMAO were measured. The AS group included 42 (36.5%) patients, and compared with the non-AS group, the rates of diabetes, hypertension, older age, systolic blood pressure, serum glucose, and TMAO levels were high. In the multivariate logistic regression analysis, serum TMAO and age were independently linked with AS after correcting for the factors significantly associated with AS. Following multivariate stepwise linear regression analysis, serum TMAO in these individuals was found to be strongly correlated with cfPWV values (*p* < 0.001). In patients on chronic HD, serum TMAO level is an independent measure of AS and strongly correlated with cfPWV.

## 1. Introduction

Cardiovascular (CV) disease is the primary cause of death of patients receiving hemodialysis (HD) for kidney failure [1,2]. Along with the more common CV risk factors, the chronic kidney disease (CKD) population including those on dialysis therapy also has certain CV risk factors, including uremic toxins, endothelial dysfunction, oxidative stress, proinflammatory cytokines, protein carbamylation, CKD-related mineral and bone disorders, and possibly intestinal dysbiosis [2]. The mixed effects of traditional and non-traditional CV risk factors result in adverse CV consequences. In addition to the influence on the myocardium, which leads to left ventricular hypertrophy and heart failure, vasculopathy including atherosclerosis and arteriosclerosis is also prominent [3]. Aortic stiffness (AS) is characterized by a decline in the compliance of the arterial walls resulting from a change in the constituents of the vessel wall [4]. Various factors are closely linked to AS, including age, sex, ethnicity, hypertension, diabetes mellitus (DM), history of cigarette smoking or alcohol consumption, dietary intake, and exercise [5,6]. Vascular stiffening is a predictor of CV and all-cause mortality in individuals on maintenance HD [7,8]. Currently, the gold-standard noninvasive measurement of AS is thought to be carotid–femoral pulse wave velocity (cfPWV) [9].

Uremic toxins accumulate in parallel with the degree of kidney failure. Several uremic toxins such as indoxyl sulfate, p-cresol sulfate, fibroblast growth factor-23, and asymmetric dimethylarginine have been found to have association or direct causal relationship with CV complications and death [10,11,12]. Trimethylamine *N*-oxide (TMAO) has biological effects and may contribute to atherosclerosis and inflammation [13]. Trimethylamine is absorbed in the intestine and then oxidized by the liver to produce TMAO [14]. It was produced by intestinal microbiota from food-derived substances such as choline, betaine, carnitine, and phosphatidylcholine. TMAO is primarily excreted by the kidneys. According to human and animal studies, TMAO is linked to CV illnesses, kidney failure, nervous system failure, and death [15,16]. Among patients with DM having CKD, a lower urine-to-plasma TMAO ratio could predict CV mortality and death from any causes, independent of underlying comorbid conditions, estimated glomerular filtration rate, and degree of albuminuria [17].

Numerous studies have examined the possible link between TMAO and AS [18,19]. Positive associations between TMAO concentrations and AS and systolic blood pressure (SBP) were seen in healthy middle-aged and older individuals, which remained independent after the adjustment of CV risk profiles; however, the significant correlation was attenuated after the use of age as adopted factor [18]. In the animal experiment, young and old mice were given chronic dietary TMAO supplementation for several months, which increased the aortic pulse wave velocity as a measure of AS [18]. A previous study found a positive correlation between serum TMAO and peripheral arterial stiffness as measured by the brachial–ankle pulse wave velocity in patients with CKD stages 3–5 [19]. This study aimed to investigate the link between AS and serum TMAO levels of patients on HD.

## 2. Results

The initial characteristics of the 115 patients undergoing HD in the present study are listed in Table 1. Among them, DM and hypertension were diagnosed in 50 (43.5%) and 61 (53.0%) individuals, respectively. A total of 42 patients (36.5%) were diagnosed with AS. The AS group was older (*p* = 0.028), had higher SBPs (*p* = 0.025), had higher glycemic levels (*p* = 0.029), and had higher serum TMAO concentrations (*p* < 0.001) than those who did not meet the criteria for AS. They also had a higher percentage of DM (*p* < 0.001) and hypertension (*p* = 0.026). Body mass index (BMI), sex, and diastolic blood pressure (DBP) were not significantly different between the two groups. The serum levels of total cholesterol, triglyceride, calcium, phosphate, and intact parathyroid hormone (iPTH) were not significantly different between the AS and non-AS groups. Indicators of dialysis adequacy including fractional clearance index for urea (Kt/V) and urea reduction ratio (URR) were also comparable between the groups. In addition, no difference was found in the percentage of participants in the two groups who used statins, fibrates, calcium channel blockers, angiotensin receptor blockers, and β-blockers.

**Figure 1:**
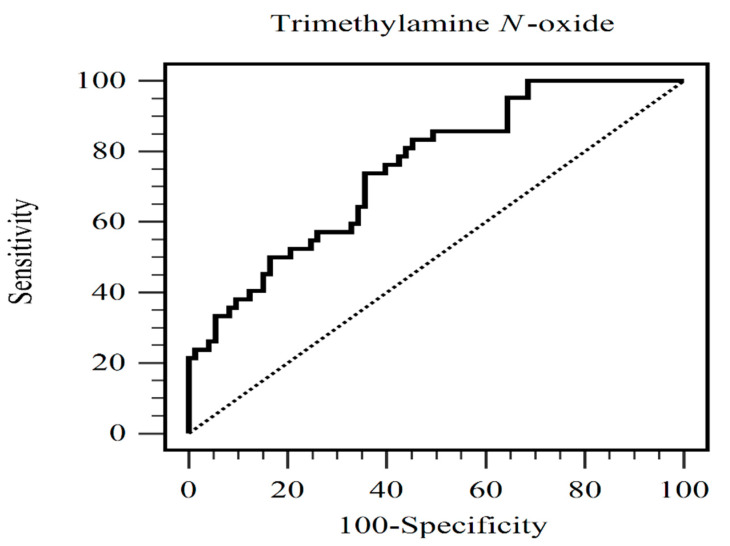
The receiver operating characteristic curve analysis of serum trimethylamine *N*-oxide levels to predict aortic stiffness in hemodialysis patients.

In this study, serum TMAO level (odds ratio (OR), 1.010; 95% confidence interval (CI), 1.003–1.017, *p* = 0.003) and older age (OR, 1.046; 95% CI, 1.007–1.088, *p* = 0.021) could independently predict AS in patients undergoing chronic HD (Table 2) using multivariate logistic regression analysis with the adjustment of clinical variables significantly correlated with AS (age, SBP, glucose, TMAO, DM, and hypertension). In addition, the area under the receiver operating characteristic (ROC) curve of serum TMAO for separating the AS group from the non-AS group was 0.751 (95% CI, 0.662–0.827; *p* < 0.001) (Figure 1). The optimal TMAO value was 118.95 μg/L, with sensitivity and specificity of 73.8% (95% CI, 58.0%–86.1%) and 64.4% (95% CI, 52.3%–75.3%), respectively. Since age is a well-known risk factor for AS, we also performed the ROC curve analysis of age to distinguish the AS group from the non-AS group (Supplementary Figure S1). The area under the ROC curve was 0.631 (95% CI, 0.536–0.719; *p* = 0.013).

Table 3 displays the relationship between cfPWV and clinical factors in individuals undergoing maintenance HD. The simple linear regression analysis revealed that DM (*r* = 0.443; *p* < 0.001), hypertension (*r* = 0.200, *p* = 0.032), age (*r* = 0.253, *p* = 0.006), SBP (*r* = 0.187, *p* = 0.046), logarithmically transformed glucose (log-glucose, *r* = 0.293, *p* = 0.001), and serum log-TMAO levels (*r* = 0.453, *p* < 0.001) positively correlated with cfPWV in these patients. DM (β = 0.320, adjusted R^2^ change = 0.108, *p* < 0.001), age (β = 0.163, adjusted R^2^ change = 0.020, *p* = 0.041), and log-TMAO levels (β = 0.355, adjusted R^2^ change = 0.198, *p* < 0.001) were independent predictors of cfPWV, according to multivariate stepwise linear regression analysis of some variables that are significantly associated with cfPWV values (including DM, hypertension, age, SBP, log-glucose, and log-TMAO). By contrast, the correlation of underlying hypertension, SBP, and log-glucose levels with cfPWV was attenuated after the multivariate linear regression.

## 3. Discussion

This study mainly found that among patients with kidney failure on maintenance HD, serum TMAO concentrations and patient age were independently linked with AS. In addition to the underlying DM, age and log-TMAO levels could predict cfPWV values in these patients.

Patients on maintenance HD are more likely to develop atherosclerotic CV diseases, which have high morbidity and mortality rates [2,20]. The pathogenesis of AS in patients on chronic HD includes vascular calcification and premature aging of the arterial wall. Prolonged inflammation, uremic toxins, high blood pressure, and changes to the vascular wall are mechanisms that mediate the processes [21,22]. In patients with HD, an observational study found a correlation between annual changes in cfPWV and older age, greater low-density lipoprotein cholesterol, lower high-density lipoprotein cholesterol, lower ankle-brachial blood pressure index, and underlying macrovascular diseases [23]. Higher levels of plasma pentosidine, an advanced glycation end product that accumulates in kidney failure, can indicate AS progression more quickly in patients on HD [24].

In our investigation focusing on individuals undergoing HD, serum TMAO levels were independently associated with AS. The atherosclerotic process is thought to be intensified by TMAO, which resulted from the metabolism of the gut microbiota [25]. The pro-atherogenic function of TMAO is explained by several pathophysiological processes. Numerous intracellular signaling pathways, including nuclear factor-κB, mitogen-activated protein kinase, and the nod-like receptor family pyrin domain containing 3 (NLRP3) inflammasome, are involved in the inflammatory damage to the vascular endothelium by TMAO [25]. Increased endothelial permeability, leukocyte adhesion, and production of proinflammatory cytokines further damage the vascular endothelium by TMAO [13,25]. Furthermore, the formation of reactive oxygen species induced oxidative stress [26]. In addition, TMAO lowers reverse cholesterol transport and cholesterol metabolism [27]. TMAO also leads to the transformation of macrophage foam cells, makes atherosclerotic plaque more vulnerable to rupture, and induces thrombogenesis [25,28]. Under the influence of TMAO, advanced glycation end-products increase, gather, and cross-link with collagen fibers in vessel walls, causing vascular stiffness [29,30]. In summary, TMAO causes AS through various molecular pathways.

Studies investigating the connection between TMAO and clinical outcomes in patients on HD have been conducted in the past few years. Among patients with HD for kidney failure, serum TMAO levels independently correlated with cardiac death and all-cause mortality [31,32]. However, TMAO did not significantly correlate with CV prognosis in patients with HD having concurrent moderate-to-severe hyperparathyroidism [33]. TMAO possibly has a pathogenic function in this disorder because it was independently correlated with the presence of protein energy wasting [34]. A small cross-sectional study found that TMAO was recognized as an independent factor for the development of abdominal aortic calcification [35]. Furthermore, TMAO appeared to be an independent indicator of the malnourished state in these patients [36]. To our knowledge, this study was the first to evaluate the correlation between TMAO and AS in patients with HD as long-term kidney replacement therapy.

In these patients undergoing HD, age has also been observed to predict AS independent of blood pressure and blood glucose levels. Aging promotes stiffening of vessels, and this increases cfPWV significantly [37]. Increasing collagen-to-elastin ratios in vessel walls, altered cytoskeletal components in vascular smooth muscle cells (i.e., vascular smooth muscle cell stiffness), remodeling of the extracellular matrix and cell–matrix crosstalk, endothelial dysfunction, and vascular calcium deposition are some of the proposed mechanisms of age-associated vascular stiffening based on both animal and human studies. Age-related AS is also predisposed by underlying comorbid diseases such DM and hypertension [38,39]. Advanced age was found to be an independent predictor of cfPWV in a previous study of patients on peritoneal dialysis [40]. In summary, in patients on HD, TMAO is probably implicated in the development of vasculopathy and serves as a sign of malnutrition and poor outcome. To the best of the authors’ knowledge, this is the first study that assessed the relationship between TMAO and AS in patients on chronic HD.

Another significant finding of this study was that DM exhibited an independent association with cfPWV. Biomarkers of AS including cfPWV, augmentation index, and brachial–ankle pulse wave velocity become abnormal even when patients have the disorders of impaired fasting glucose or impaired glucose tolerance [41]. Higher pulse wave velocities were associated with both microvascular and macrovascular problems in patients with DM [42]. DM-related AS pathogenesis is complex. In DM, advanced glycation end products are overproduced and cross-link with collagen fibers in the vessel walls, reducing the elastic properties of the blood vessels and increasing vascular stiffness [42,43]. In addition, insulin resistance contributes to AS by causing endothelial dysfunction, immunodeficiency, and abnormal renin–angiotensin–aldosterone system activation [43,44].

This study has several limitations. First, given the cross-sectional design, determining whether TMAO was directly responsible for the vascular stiffness was challenging. Second, the sample size was small. Third, whether the subgroup with kidney failure would benefit from the existing cfPWV cutoff of 10 m/s used to differentiate between AS and non-AS was unknown. Fourth, as meals derived from animals and alcohol may alter serum TMAO levels, dietary consumption was not evaluated. To further support the findings of this study, larger studies with a longitudinal design are warranted.

## 4. Conclusions

Aside from DM and older age, an increase in serum TMAO concentration was proved to be an independent predictor of AS among patients on HD.

## 5. Materials and Methods

### 5.1. Patients

From 1 March 2015 to 30 June 2015, 115 patients on maintenance HD were recruited for the cross-sectional study at the HD unit of a hospital in Hualian, Taiwan. Participants were evaluated for eligibility if they were 20 years of age or older and had completed a typical 4-h HD session three times per week for at least 6 months. Dialysis was carried out using high-flow polysulfone disposable dialyzers (FX class dialyzer; Fresenius Medical Care, Bad Homburg, Germany). Based on the medical records, information was gathered on basic demographic characteristics and medical history, such as DM and hypertension. The Research Ethics Committee of the hospital approved the study, which followed the general guidelines of the Declaration of Helsinki (IRB108-96-B). The study excluded patients who refused to participate or who had a history of limb amputation, an active infection, cancer, heart failure, or were bedridden.

### 5.2. Anthropometric Analysis and Biochemical Investigations

BMI was determined by dividing post-HD weight (kg) by height squared (m^2^) after the participants’ heights and post-HD weights were assessed. From each patient, 5 mL of blood was drawn before dialysis. An autoanalyzer (SiemensAdvia 1800, Siemens Healthineers Headquarters, Siemens Healthcare GmbH, Henkestr, Erlangen, Germany) was used to measured serum levels of blood urea nitrogen (BUN), creatinine, albumin, total cholesterol, triglyceride, glucose, calcium, and phosphorus. When the pre- and post-dialysis BUN levels were measured, a formal, single-compartment dialysis urea kinetic model was used to determine the Kt/V and URR. A commercially available enzyme-linked immunosorbent test kit (Diagnostic Systems Laboratories, Webster, TX, USA) was used to assess serum iPTH levels [45].

### 5.3. High-Performance Liquid Chromatography (HPLC)—Mass Spectrometry for TMAO

A serum sample of 100 µL was transferred to a 1.5-mL Eppendorf tube and then mixed with 100 µL of 50 mM sodium phosphate dibasic heptahydrate solution. Moreover, 100 µL of deuterium-labeled internal standard solution (300 ng/mL d_9_-TMAO) in methanol was added and softly vortex-mixed. Then, the Simplified Liquid Extraction method (Phenomenex, Novum) with 1.5 mL of ethyl acetate as elute was used. Nitrogen was then used to make samples dry, and they were reconstituted in 100 µL of methanol for subsequent analysis. A Waters e2695 Separations Module HPLC system consisted of a single quadrupole mass spectrometer (ACQUITY QDa, Waters Corporation, Milford, MA, USA) for the measurement of serum TMAO levels. Phenomenex Luna C18(2) HPLC Column (particle size 5 µm, dimension 250 × 4.60 mm^2^, pore size 100 Å) was applied, with temperature of 40 °C, flow rate of 0.8 mL/min, and an injection volume of 30 µL. The mobile phase followed a gradient elution profile, which was administered as described below. The initial composition (95% component A, water 0.1% formic acid/95% component B, methanol 0.1% formic acid) was kept constant for 1 min. Within the subsequent 12 min, solvent B was increased in a linear manner up to 70% in proportion and then kept at 70% for 2 min. Regarding the column re-equilibration, the gradient of solvent B decreased to 50% in 1 min and persisted in the next 2 min. A modified approach was used to examine HPLC mass spectrometry [46]. With all the samples being pre-treated, quantification was simultaneously determined in the positive-ion mode (TMAO) with the assistance of electrospray ionization (ESI). The parameters were set as follows: desolvation temperature of 600 °C, capillary voltage of 800 V, and cone voltage of 15.0 V. Mass spectrometry was employed with complete scan ranges of 50–450 *m*/*z* for positive-ion modes and 100–350 *m*/*z* for negative-ion modes to monitor the participant’s compound (TMAO: 76.0 *m*/*z*; d9-TMAO: 85.1 *m*/*z*). The retention times for TMAO and d_9_-TMAO were 2.54 min. All examinations were gathered and analyzed using the Empower^®^ 3.0 application (New York, NY, USA).

### 5.4. Blood Pressure and Aortic Stiffness Measurements

SBP and DBP were measured three times at 5-min intervals before HD after a 30-min period of rest. Pressure applanation tonometry (SphygmoCor system, AtCor Medical, Sydney, New South Wales, Australia) was used to measure the cfPWV to determine AS [45]. Before cfPWV measurements, patients sat quietly for at least 10 min in a room with consistent temperature while lying flat. According to a clinical practice guideline [47], the AS group had cfPWV values >10 m/s; those with a value of <10 m/s were assigned to the non-AS group.

### 5.5. Statistical Analysis

To determine whether the variables had a normal distribution, the Kolmogorov–Smirnov test was used. The mean and standard deviation (SD) of normally distributed data were used to compare the means of the two groups using Student’s independent *t*-test (two-tailed). We used the Mann–Whitney U test to compare the results between the groups for variables (HD duration, triglyceride, glucose, iPTH, and TMAO) that were not normally distributed; these data were transformed to logarithms (base 10) to achieve normality. Categorical variables were presented as numbers and percentages, and the two test was used to compare the two groups. Multivariate logistic regression analysis was used to further assess variables substantially related to AS. A simple linear regression analysis was used to determine the relationship between clinical factors and cfPWV values in patients on chronic HD, and variables that showed a significant relationship were then checked for independence using a multivariate forward stepwise regression analysis. The area under the curve was calculated using receiver operating characteristic analysis to establish the ideal TMAO levels for AS prediction in patients on HD. IBM SPSS for Windows version 19.0 (IBM Corp., Armonk, NY, USA) was used to analyze the data. A *p* value < 0.05 was regarded as statistically significant.

## Figures and Tables

**Table 1:** Baseline characteristics of the 115 chronic hemodialysis patients with non-aortic stiffness group (cfPWV ≤ 10.0 m/s) or aortic stiffness group (cfPWV > 10.0 m/s).

Characteristics	All Patients (*n* = 115)	Group without Aortic Stiffness (*n* = 73)	Aortic Stiffness Group (*n* = 42)	*p* Value
Age (years)	63.10 ± 13.46	61.01 ± 14.06	66.71 ± 11.64	0.028 *
HD duration (months)	59.88 (24.72–130.08)	80.40 (25.14–143.10)	47.34 (24.30–83.01)	0.068
Height (cm)	159.97 ± 8.44	159.77 ± 8.92	160.33 ± 7.62	0.731
Pre-HD body weight (kg)	63.06 ± 15.22	62.31 ± 15.65	64.38 ± 14.53	0.485
Post-HD body weight (kg)	60.87 ± 14.77	60.17 ± 15.16	62.09 ± 14.15	0.505
Body mass index (kg/m^2^)	24.66 ± 4.81	24.41 ± 4.99	25.10 ± 4.49	0.465
Carotid–femoral PWV (m/s)	9.43 ± 2.20	7.99 ± 0.99	11.92 ± 1.30	<0.001 *
Systolic blood pressure (mm Hg)	142.30 ± 25.16	138.33 ± 24.15	149.21 ± 25.66	0.025 *
Diastolic blood pressure (mm Hg)	76.50 ± 16.28	77.51 ± 16.63	74.76 ± 15.69	0.386
Albumin (g/dL)	4.14 ± 0.44	4.14 ± 0.44	4.15 ± 0.46	0.890
Total cholesterol (mg/dL)	144.96 ± 35.19	145.42 ± 38.31	144.14 ± 29.39	0.852
Triglyceride (mg/dL)	112.00 (87.00–178.00)	106.00 (83.50–181.50)	122.00 (90.75–174.25)	0.292
Glucose (mg/dL)	135.00 (110.00–172.00)	132.00 (103.50–159.00)	140.00 (122.00–200.00)	0.029 *
Blood urea nitrogen (mg/dL)	60.17 ± 14.04	58.95 ± 13.55	62.31 ± 14.78	0.218
Creatinine (mg/dL)	9.25 ± 2.07	9.37 ± 2.08	9.05 ± 2.07	0.437
Total calcium (mg/dL)	8.96 ± 0.75	8.93 ± 0.75	9.03 ± 0.76	0.477
Phosphorus (mg/dL)	4.62 ± 1.29	4.61 ± 1.30	4.65 ± 1.28	0.856
iPTH (pg/mL)	185.90 (58.50–372.50)	244.40 (97.40–413.15)	114.30 (51.88–312.10)	0.117
TMAO (μg/L)	118.95 (75.92–198.21)	94.32 (61.69–159.79)	178.81 (114.09–273.52)	<0.001 *
Urea reduction rate	0.74 ± 0.04	0.74 ± 0.05	0.73 ± 0.04	0.731
Kt/V (Gotch)	1.35 ± 0.17	1.35 ± 0.18	1.33 ± 0.14	0.567
Female, *n* (%)	58 (50.4)	38 (52.1)	20 (47.6)	0.647
Diabetes mellitus, *n* (%)	50 (43.5)	21 (28.8)	29 (69.0)	<0.001 *
Hypertension, *n* (%)	61 (53.0)	33 (45.2)	28 (66.7)	0.026 *
Coronary artery disease, *n* (%)	72 (62.6)	43 (58.9)	29 (69.0)	0.281
Angiotensin receptor blocker, *n* (%)	33 (28.7)	19 (26.0)	14 (33.3)	0.404
β-blocker, *n* (%)	40 (34.8)	23 (31.5)	17 (40.5)	0.331
Calcium channel blocker, *n* (%)	45 (39.1)	30 (41.1)	15 (35.7)	0.569
Statin, *n* (%)	19 (16.5)	9 (12.3)	10 (23.8)	0.110
Fibrate, *n* (%)	12 (10.4)	7 (9.6)	5 (11.9)	0.696

Values for continuous variables are shown as mean ± standard deviation after analysis by Student’s *t*-test; variables not normally distributed are shown as median and interquartile range after analysis by the Mann–Whitney U test; values are presented as number (%) and analysis after analysis by the chi-square test. HD, hemodialysis; PWV, pulse wave velocity; iPTH, intact parathyroid hormone; TMAO, trimethylamine *N*-oxide; Kt/V, fractional clearance index for urea. * *p* < 0.05 was considered statistically significant.

**Table 2:** Multivariate logistic regression analysis of the factors correlated to aortic stiffness.

Variables	Odds Ratio	95% Confidence Interval	*p* Value
TMAO, 1 μg/L	1.010	1.003–1.017	0.003 *
Age, 1 year	1.046	1.007–1.088	0.021 *
Glucose, 1 mg/dL	1.004	0.995–1.012	0.441
Systolic blood pressure, 1 mm Hg	1.017	0.993–1.041	0.162
Diabetes mellitus (present)	2.771	0.921–8.337	0.070
Hypertension (present)	1.832	0.635–5.283	0.262

TMAO, trimethylamine *N*-oxide. Analysis data were carried out using the multivariate logistic regression analysis (adopted factors: diabetes mellitus, hypertension, age, systolic blood pressure, glucose, and TMAO). * *p* < 0.05 was considered statistically significant.

**Table 3:** Correlation between carotid—femoral pulse wave velocity levels and clinical variables.

Variables	Carotid–Femoral Pulse Wave Velocity (m/s)				
	Simple Linear Regression		Multivariate Linear Regression		
	*r*	*p* Value	Beta	Adjusted R^2^ Change	*p* Value
Female	−0.041	0.663	-	-	-
Diabetes mellitus	0.443	<0.001 *	0.320	0.108	<0.001 *
Hypertension	0.200	0.032 *	-	-	-
Age (years)	0.253	0.006 *	0.163	0.020	0.041 *
Log-HD duration (months)	−0.119	0.205	-	-	-
Height (cm)	0.091	0.333	-	-	-
Pre-HD body weight (Kg)	0.124	0.186	-	-	-
Body mass index (Kg/m^2^)	0.112	0.234	-	-	-
Systolic blood pressure (mm Hg)	0.187	0.046 *	-	-	-
Diastolic blood pressure (mm Hg)	0.063	0.504	-	-	-
Albumin (g/dL)	0.107	0.254	-	-	-
Total cholesterol (mg/dL)	−0.023	0.806	-	-	-
Log-triglyceride (mg/dL)	0.038	0.687	-	-	-
Log-glucose (mg/dL)	0.293	0.001 *	-	-	-
Blood urea nitrogen (mg/dL)	0.123	0.191	-	-	-
Creatinine (mg/dL)	0.022	0.813	-	-	-
Total calcium (mg/dL)	0.053	0.571	-	-	-
Phosphorus (mg/dL)	0.114	0.223	-	-	-
Log-iPTH (pg/mL)	−0.119	0.206	-	-	-
Log-TMAO (μg/L)	0.453	<0.001 *	0.355	0.198	<0.001 *
Urea reduction rate	−0.102	0.277	-	-	-
Kt/V (Gotch)	−0.113	0.230	-	-	-

Data on HD duration, triglyceride, glucose, iPTH, and TMAO levels showed skewed distribution, and therefore were log-transformed before analysis. Analysis data were carried out using the simple linear regression analyses or multivariate stepwise linear regression analysis (adopted factors: diabetes mellitus, hypertension, age, systolic blood pressure, log-glucose, and log-TMAO). HD, hemodialysis; iPTH, intact parathyroid hormone; TMAO, trimethylamine *N*-oxide; Kt/V, fractional clearance index for urea. * *p* < 0.05 was considered statistically significant.

## Data Availability

The data presented in this study are available on request from the corresponding author.

## Supplementary Materials

The following supporting information can be downloaded at: https://www.mdpi.com/article/10.3390/toxins15090572/s1, Figure S1: The receiver operating characteristic curve analysis of age in the prediction of aortic stiffness in patients on maintenance hemodialysis.
