# Supplementary material for: Serum Trimethylamine N-Oxide Level Is Positively Associated with Aortic Stiffness Measured by Carotid–Femoral Pulse Wave Velocity in Patients Undergoing Maintenance Hemodialysis
Source: Toxins (Basel). 2023 Sep 17;15(9):572. doi: 10.3390/toxins15090572 (PMC10538077; doi:10.3390/toxins15090572)
Supplement: Supplementary file 1 [file toxins-15-00572-s001.zip › toxins-2579815-supplementary.pdf]

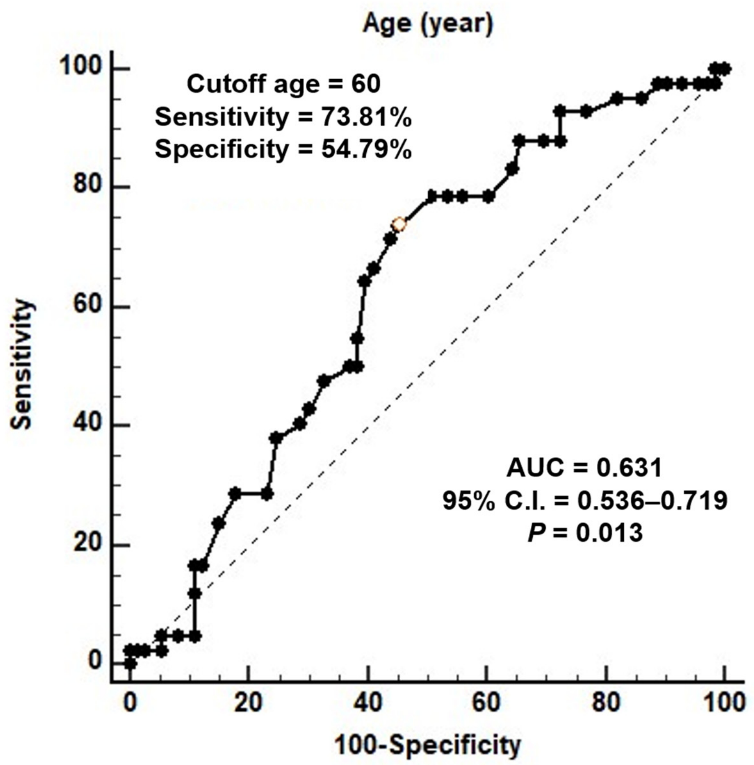

Figure S1. The receiver operating characteristic curve analysis of age in the prediction of aortic stiffness in patients on maintenance hemodialysis.
